# Teamwork Optimization Algorithm: A New Optimization Approach for Function Minimization/Maximization

**DOI:** 10.3390/s21134567

**Published:** 2021-07-03

**Authors:** Mohammad Dehghani, Pavel Trojovský

**Affiliations:** 1 Department of Electrical and Electronics Engineering, Shiraz University of Technology, Shiraz 71557-13876, Iran; m.dehghani@sutech.ac.ir; 2 Department of Mathematics, Faculty of Science, University of Hradec Králové, 500 03 Hradec Králové, Czech Republic

**Keywords:** optimization, optimization algorithm, optimization problem, population-based, teamwork

## Abstract

Population-based optimization algorithms are one of the most widely used and popular methods in solving optimization problems. In this paper, a new population-based optimization algorithm called the Teamwork Optimization Algorithm (TOA) is presented to solve various optimization problems. The main idea in designing the TOA is to simulate the teamwork behaviors of the members of a team in order to achieve their desired goal. The TOA is mathematically modeled for usability in solving optimization problems. The capability of the TOA in solving optimization problems is evaluated on a set of twenty-three standard objective functions. Additionally, the performance of the proposed TOA is compared with eight well-known optimization algorithms in providing a suitable quasi-optimal solution. The results of optimization of objective functions indicate the ability of the TOA to solve various optimization problems. Analysis and comparison of the simulation results of the optimization algorithms show that the proposed TOA is superior and far more competitive than the eight compared algorithms.

## 1. Introduction

Optimization is the setting of inputs or specifications of a device, a mathematical function, or an experiment in which the output (result) is maximized or minimized [1,2]. In optimization problems, the input contains a number of parameters (decision variables), the process or function is called the cost function or the fitness function, and the output is called the cost or fitness [3]. An optimization problem with only one variable is a one-dimensional optimization problem, otherwise it is a multidimensional optimization problem. As the dimensional of the optimization problem increases, optimization will become more difficult [4].

In general, optimization problem-solving methods are classified into two classes of deterministic and stochastic methods [5]. Deterministic methods include two groups of gradient-based methods and non-gradient based methods. Methods that use derivative information or gradients of objective functions and constraint functions are called gradient-based methods. These methods are divided into two categories of first-order and second-order gradient methods. The first-order gradient methods use only the objective function gradient and constraint functions, while the second-order gradient methods use Hessian information of the objective function or constraint functions in addition to the gradient [6]. One inherent drawback of mathematical programming methods is a high probability of stagnation in local optima during the search of non-linear space. Methods that do not use gradients or Hessian information in any way (either analytically or numerically) are called non-gradient-based or zero-order methods. One of the drawbacks of non-gradient-based deterministic methods is that their implementation is not easy and requires high mathematical preparation to understand and use them.

Population-based optimization algorithms (PBOAs) are stochastic methods that are able to provide appropriate solutions to optimization problems without the need for derivative information and gradients and are based on random scanning of the problem search space [7]. PBOAs are methods that first offer a number of random solutions to an optimization problem. Then, in an iterative process based on the steps of the algorithm, these proposed solutions are improved. After completing the iterations of the algorithm and at the end of implementing the algorithm on the optimization problem, the algorithm presents the best proposed solution for the optimization problem.

Each optimization problem has a basic solution called a global optimal solution. Achieving a global optimal solution requires a lot of time and computation in some complex optimization problems. The advantage of PBOAs is that they provide suitable solutions in less time and computations. The important issue about solutions provided by PBOAs is that these solutions are not necessarily global optimal solutions. For this reason, the solution provided by the PBOAs is called a quasi-optimal solution. The quasi-optimal solution is at best the same as the global optimal, otherwise it should be as close to the global optimal as possible. Therefore, in comparing the performance of several PBOAs in solving an optimization problem, the algorithm that presents the quasi-optimal solution closer to the global optimal is a better algorithm. Additionally, an optimization algorithm may perform well in solving one optimization problem but fail to solve another optimization problem and fail to provide a suitable quasi-optimal solution. For these reasons, the various PBOAs have been proposed to solve optimization problems and provide more appropriate quasi-optimal solutions.

PBOAs are presented based on modeling various processes and phenomena such as behaviors of animals, plants, and other living organisms in nature, laws of physics, rules of games, genetics and reproductive processes, and any other phenomenon or process that has the character of an evolutionary process or progress. For example, simulation of natural ant behaviors has been used in the design of Ant Colony Optimization (ACO) [8]. Hook’s physical law simulation has been used to design the Spring Search Algorithm (SSA) [9]. The rules of the game of throwing a ring and simulating the behavior of the players have been used in the design of Ring Toss Game-Based Optimization (RTGBO) [1]. Immune system simulation is used in the design of the Artificial Immune System algorithm as an evolutionary algorithm [10]. Additionally, by ideation of different phenomena, it is possible to design different systems and algorithms for problem solving. For example, in the Blackboard system, which is an artificial intelligence approach based on the Blackboard architecture model, a common knowledge base, the “blackboard”, is repeatedly updated by a diverse group of specialized knowledge resources [11].

The Genetic Algorithm (GA) is one of the most widely used and oldest evolutionary-based optimization methods which has been developed based on simulation of reproductive processes and Darwin’s theory of evolution. In the GA, changes in chromosomes occur during the reproductive process. Parents’ chromosomes are first identified by a selection operator, then randomly exchanged through a special process called crossover. Thus, children inherit some of the traits or characteristics of the father and some of the traits or characteristics of the mother. A rare process called mutation also causes changes in the characteristics of living things. Finally, these new children are considered as parents in the next generation and the process of the algorithm is repeated until the end of the implementation of the algorithm [12]. The concepts of the GA are simple and understandable, but having control parameters and high computation are the main disadvantages of the GA.

Particle Swarm Optimization (PSO) is another old and widely used method of optimization that is inspired by the natural behaviors of birds and the relationships between them. In PSO, the position of each member of the population is updated at any point in time under the influence of three components. The first component is the speed of the population member in the previous repetition, the second component is the personal experience of the population member gained until each repetition, and the third component is the experience that the entire population has gained up to that repetition. [13]. One of the advantages of PSO is the simplicity of the relationship and its implementation. However, poor convergence and entrapment in local optimum areas are the main disadvantages of PSO.

The Gravitational Search Algorithm (GSA) is a physics-based optimization algorithm based on modeling the gravitational force between objects. In the GSA, members of the population are objects that are in the problem-solving space at different distances from each other. Objects that have a more optimal position in the search space attract other objects based on the gravitational force [14]. High computation, a time-consuming process, having several control parameters, and poor convergence in complex objective functions are the most important disadvantages of the GSA.

Teaching–Learning-Based Optimization (TLBO) is an intelligent optimization algorithm inspired by the learning and teaching process between a teacher and students in the classroom. In the TLBO algorithm, a mathematical model for teaching and learning is considered, which is finally implemented in two stages and can lead to optimization:

(i) Teaching stage: In this stage, the best member of the population is selected as a teacher and directs the average population towards itself. This is similar to what a teacher really does in the real world.
(ii) Learning phase: In this phase, people in the population (who are considered classmates) develop their knowledge by cooperation together. This is similar to what really happens in relationships between friends and classmates [15].

The main disadvantage of TLBO is the convergence rate, and it gets even worse when dealing with high-dimensional problems.

The Grey Wolf Optimizer (GWO) is a swarm-based algorithm which models the hierarchical structure and social behavior of gray wolves while hunting. In the GWO, four types of gray wolves, alpha, beta, delta, and omega, are used to simulate the leadership hierarchy. In addition, the process of hunting is mathematically modeled in three main stages of searching prey, encircling prey, and attacking prey. In the GWO, alpha is the best solution, and beta and delta are the second and third best solutions. Other members of the population are considered as omega wolves. The hunting process is led by the alpha, beta, and delta wolves, while omega wolves follow these three types of wolves [16]. The main disadvantages of the GWO include low convergence speed, poor local search, and low accuracy in solving complex problems.

The Whale Optimization Algorithm (WOA) is a nature-inspired meta-heuristic optimization algorithm which mimics the social behavior of humpback whales. In the WOA, the bubble-net hunting strategy is simulated in three stages of encircling prey, spiral bubble-net feeding maneuver, and search for prey [17]. Low accuracy, slow convergence, and easily falling into local optimum are the main disadvantages of the WOA. Moreover, the WOA cannot perform well enough in solving high-dimensional optimization problems.

The Marine Predators Algorithm (MPA) which is a swarm-based algorithm which is inspired by the movement strategies that marine predators use when trapping their prey in the ocean. The optimization process in the MPA is divided into three main optimization stages due to the different hunting and prey speeds (see [18]):

Phase 1: When the prey moves faster than the hunter.
Phase 2: When the prey and the hunter move at almost the same speed.
Phase 3: When the hunter is moving faster than the prey.

High computation, a time-consuming process, and having two control parameters whose adjustment is an important challenge in the quality of performance of the MPA are the most important disadvantages of the MPA.

The Tunicate Swarm Algorithm (TSA) is a swarm-based approach which is based on the simulation of swarm behaviors and jet propulsion of tunicates pending the navigation and foraging process. Although a tunicate has no mindset or idea about food sources, it is able to find food sources. In the TSA, jet propulsion behavior is modeled based on avoiding collisions between population members, moving toward the best member, and staying close to it. Swarm behavior has also been used to update members of the population [19].

Poor convergence and falling to local optimal solutions in solving high-dimensional multimodal optimization problems are the main disadvantages of the TSA. In addition, the TSA has several control parameters, and assigning appropriate values for these parameters is a challenging process.

The innovation and contribution of this paper is in presenting a new population-based optimization algorithm called the Teamwork Optimization Algorithm (TOA) to solve various optimization problems. The main idea in designing the proposed TOA is to simulate the activities, behaviors, and interactions of team members in performing teamwork in order to achieve the goal of the team. The theory and various stages of the TOA are explained and then, to implement it in optimization problems, its mathematical modeling is also presented. The capability of the TOA in optimizing and presenting suitable quasi-optimal solutions on a standard set of different objective functions is evaluated. In order to analyze and evaluate the quality of the obtained optimization results, the performance of the TOA is compared with eight well-known algorithms.

The rest of this paper is organized as follows. The proposed TOA is presented in Section 2. Simulation studies and performance analysis of the TOA are presented in Section 3. A discussion of the performance of the TOA is presented in Section 4. Finally, conclusions and several suggestions for future studies are presented in Section 5.

## 2. Teamwork Optimization Algorithm

In this section, the theory of the proposed algorithm is stated and its mathematical model is presented in order to implement it in solving optimization problems.

The Teamwork Optimization Algorithm (TOA) is a PBOA which is designed based on simulation of relationships and behaviors of team members in performing their duties and achieving the desired goal of the team. Therefore, in the TOA, the search agents are team members, the relationships between team members are a tool for transmitting information, and the goal of the team is actually the solution to the optimization problem.

In a team that uses teamwork to achieve a common goal, the relationships and behaviors of team members can be considered as follows:

Supervisor: A member of the team who is responsible for leading and guiding the team. The supervisor is the member of the team who has the best performance compared to the other team members.

Team members: Other members who perform more weakly than the supervisor.

Influence of supervisor on team members: Each team member strives to improve their performance in accordance with the supervisor’s guidelines and instructions.

Influence of better members on weak team members: Each team member tries to improve her/his performance by using the experiences of other team members who perform better than themselves.

Individual activities: Each team member, based on personal efforts and activities, tries to contribute more to the achievement of the whole team.

Modeling of the mentioned concepts is applied in the design of the proposed TOA.

In the proposed TOA, each member of the population represents a proposed solution to the optimization problem. In fact, each member of the population proposes values for the problem variable. This means that each member of the population can be mathematically modeled as a vector whose number of components is equal to the number of problem variables. Therefore, the population of the algorithm using a matrix whose number of rows is equal to the number of members of the population and the number of columns is equal to the problem variables and can be represented by Equation (1).

(1) $$X={\left[\begin{array}{c} \begin{array}{c} X_{1}\\ \vdots \\ X_{i} \end{array}\\ \vdots \\ X_{N} \end{array}\;|\begin{array}{ccccc} x_{1,1} & \cdots & x_{1,d} & \cdots & x_{1,m}\\ \vdots & \ddots & \vdots & ⋰ & \vdots \\ \;x_{i,1} & \cdots & x_{i,d} & \cdots & x_{i,m}\\ \vdots & ⋰ & \vdots & \ddots & \vdots \\ x_{N,1} & \cdots & x_{N,d} & \cdots & x_{N,m} \end{array}\right]}_{N\times m},$$

where X is the population matrix of the TOA, $\;X_{i}$ is the ith team member,$\;x_{i,d}$ is the value for the dth problem variable suggested by the ith team member, N is the number of team members, and m is the number of problem variables.

As mentioned, each member of the algorithm population proposes values for the problem variables, and by placing these proposed values in the variables of the objective function, a value for the objective function is obtained. Therefore, based on each member of the population, a value is evaluated for the objective function. The vector of the objective function values is determined using the following equation:

(2) $$F={\left[\begin{array}{c} \begin{array}{c} F_{1}\\ \vdots \\ F_{i} \end{array}\\ \vdots \\ F_{N} \end{array}\;|\begin{array}{c} \begin{array}{c} F\left(X_{1}\right)\\ \vdots \\ F\left(X_{i}\right) \end{array}\\ \vdots \\ F\left(X_{N}\right) \end{array}\right]}_{N\times 1},$$

where F is the vector of the objective function and $F_{i}$ is the objective function value of the ith team member.

In each iteration of the algorithm, based on the comparison of the values of the objective function, the member who has provided the best performance among the team members is selected as the supervisor. The task of the supervisor in teamwork is to lead the team and guide the team members in order to achieve the goal of that team. The selection of the supervisor in the proposed TOA is modeled using Equation (3).

*Supervisor: S = X_s_* and *s* is the row number of the team member with a minimum of *F* vectors, (3)

where *S* is the supervisor of the team.

The algorithm population is updated in the TOA in three stages.

Stage 1: Supervisor guidance

In the first stage, team members are updated based on the supervisor’s instructions. At this stage, the supervisor shares her/his information and reports to other team members and guides them towards achieving the goal. This step of the update is simulated in the TOA using Equations (4)–(6).

(4) $$X_{i}^{S1}:x_{i,d}^{S1}=x_{i,d}+r\times \left(S_{d}-I\times x_{i,d}\right)\;,$$

(5) $$X_{i}=\{\begin{array}{c} X_{i}^{S1},\;\;F_{i}^{S1}<F_{i}\\ X_{i},\;\;else \end{array},$$

(6) I=round(1+r),

where $X_{i}^{S1}$ is the new status for the *i*th team member based on supervisor guidance, $F_{i}^{S1}$ is the objective function value, $x_{i,d}^{S1}$ is the new value for the dth problem variable suggested by the ith team member updated based on supervisor guidance, I is the update index, and r is a random number in a [0, 1] interval.

Stage 2: Information sharing

In the second stage, each team member tries to use the information of other team members who have performed better than themselves in order to improve the performance. This stage of updating team members in the proposed TOA is modeled using Equations (7)–(9).

(7) $$X^{M,i}:x_{d}^{M,i}=\frac{\sum_{j=1}^{N_{i}}x_{j,d}^{g,i}}{N_{i}}\;,$$

(8) $$X_{i}^{S2}:x_{i,d}^{S2}=x_{i,d}+r\times \left(x_{d}^{M,i}-I\times x_{i,d}\right)\times sign\left(F_{i}-F^{M,i}\right)\;,$$

(9) $$X_{i}=\{\begin{array}{c} X_{i}^{S2},\;\;F_{i}^{S2}<F_{i}\\ X_{i},\;\;else \end{array},$$

where $X^{M,i}$ is the average of the team member which is better than that of the ith team member, $F^{M,i}$ is the its objective function value, $N_{i}$ is the number of team members with better performance than the ith team member, $x_{j,d}^{g,i}$ is the value of the dth variable suggested by the jth better team member for the ith team member, $X_{i}^{S2}$ is the new status for the ith team member based on the second stage, and $F_{i}^{S2}$ is the its objective function value.

Stage 3: Individual activity

At this stage, each team member tries to improve her/his performance based on her/his current situation. This stage of updating team members is modeled using Equations (10) and (11).

(10) $$X_{i}^{S3}:x_{i,d}^{S3}=x_{i,d}+\left(-0.01+r\times 0.02\right)\times x_{i,d},$$

(11) $$X_{i}=\{\begin{array}{c} X_{i}^{S3},\;F_{i}^{S3}<F_{i}\\ X_{i},\;else, \end{array},$$

where $X_{i}^{S3}$ is the new status for the ith team member based on the third stage and $F_{i}^{S3}$ is the objective function value.

**Figure 1:**
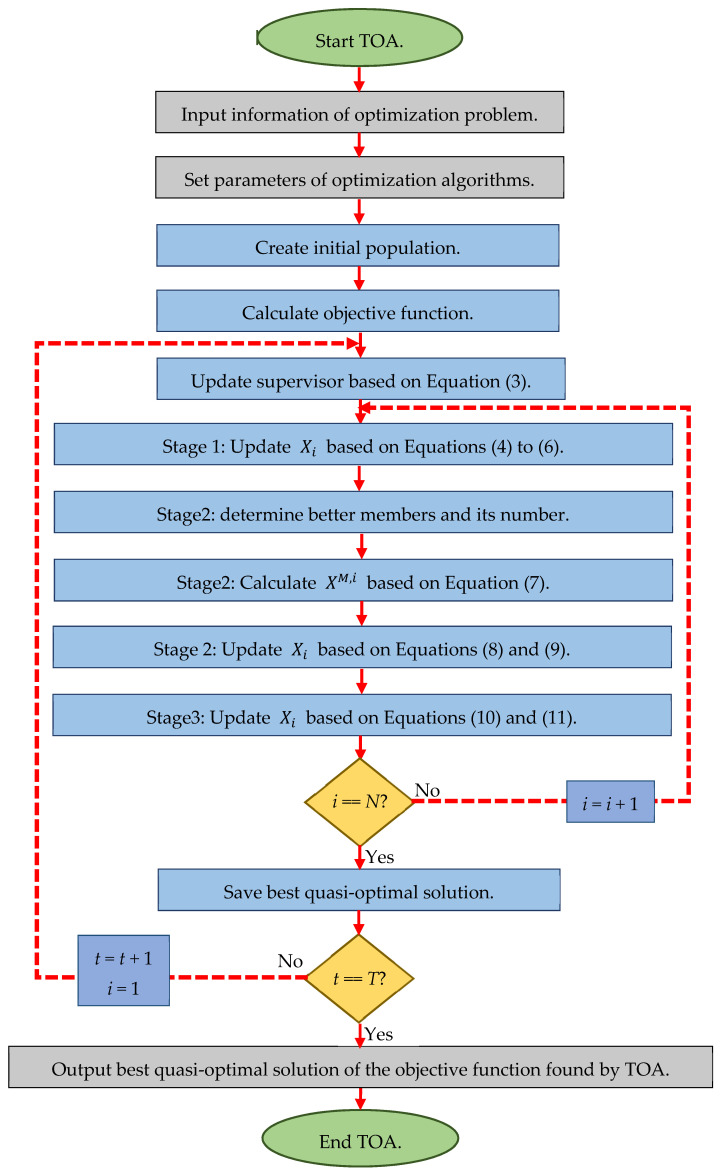
Flowchart of the proposed TOA.

In each iteration of the algorithm, the members of the population are updated in three stages according to Equations (4)–(11). The update process is repeated until the algorithm reaches the condition of stopping. Finally, after the full implementation of the algorithm, the TOA proposes the best quasi-optimal solution obtained for the optimization problem. The pseudocode of the proposed TOA is presented in Algorithm 1 and its flowchart in Figure 1.
**Algorithm 1.** Pseudocode of TOA.***Start TOA.****1.**Input problem information: variables, objective function, and constraints.**2.**Set number of team members (N) and iterations (T).**3.**Generate an initial population matrix at random.**4.**Evaluate the objective function.**5.* ***For t = 1:T****6.*  *Update supervisor based on Equation (3).**7.*   ***For i = 1:N****8.*    ***Stage1: supervisor guidance****9.*     *Update*$X_{i}$*based on first stage using Equations (4) and (6).**10.*    ***Stage2: information sharing****11.*     *Determine better team members and*$N_{i}$*for i’th team member.**12.*     *Calculate based on Equation (7).**13.*     *Update*$X_{i}$*based on second stage using Equations (8) and (9).**14.*    ***Stage 3: individual activity****15.*     *Update*$X_{i}$*based on third stage using Equations (10) and (11).**16.*   ***End For i = 1:N****17.*  *Save best quasi-optimal solution obtained with the TOA so far.**18.*
***End For t = 1:T****19.**Output best quasi-optimal solution obtained with the TOA.****End TOA.***

### Step-by-Step Example

In this subsection, the steps of the TOA are implemented and described on an objective function. For this purpose, the sphere function with 2 variables and 10 population members has been used.

Sphere function: $F(X)=F(x_{1},x_{2})=x_{1}^{2}+x_{2}^{2}\;subject\;to:-100\leq x_{1},x_{2}\leq 100$

Step 1:

In the first step, members of the population are randomly created. The values proposed for the problem variables must be within an acceptable range. The following general formula can be used to create initial and feasible random solutions:

$X_{i}:x_{d}=x_{lo}+rand\times (x_{hi}-x_{lo})\;where\;i\;=\;1:N,d\;=\;1$

*X*_1_:

$x_{1}=-100+rand\times \left(100-\left(-100\right)\right):\;x_{1}=98.12666$

$x_{2}=-100+rand\times \left(100-\left(-100\right)\right):\;x_{2}=72.68003$

Step 2:

In the second step, the objective function is evaluated based on the values proposed by each member of the population for the variables.

$F_{i}=F\left(X_{i}\right)=F\left(x_{1}:x_{d}\right)$

$F_{1}=F\left(X_{1}\right)=F\left(98.12666,72.68003\right)=14911.23$

Step 3:

In the third step, based on the comparison of the values of the objective functions, the team supervisor is determined. The supervisor is the member of the population that provides the least amount of objective function.

S: Member with the minimum value of the objective function

$S=X_{5}:\;\left[14.63504\;,\;17.21673\right]\;,\;F_{5}=510.6002$

Step 4:

In the fourth step, the members of the population are updated according to the supervisor’s instructions. This step is calculated using Equations (4)–(6).

Step 5:

In the fifth step, each member of the population is updated based on the guidance of better qualified team members. This step is calculated using Equations (7)–(9).

Step 6:

In the sixth step, each member of the population improves her/his condition with individual activities. This step is calculated using Equations (10) and (11).

Step 7:

The third to sixth steps are repeated until the stop condition is reached. Additionally, after the algorithm is fully implemented, the best solution is available.

The various steps of the proposed TOA for the first iteration are presented in Table 1. Additionally, the final solution for the “sphere function” is presented after 50 repetitions.

## 3. Simulation Studies

In this section, simulation studies on the proposed TOA performance in solving optimization problems and the ability to provide quasi-optimal solutions are presented. To achieve this goal, the performance of the TOA is implemented and analyzed on twenty-three of different types of unimodal and multimodal standard objective functions. Complete information on these objective functions and their details are provided in Appendix A and Table A1, Table A2 and Table A3 [20].

In order to analyze the quality of the results obtained from the proposed algorithm, these results have been compared with the performance of eight other well-known algorithms: Particle Swarm Optimization (PSO) [13], the Genetic Algorithm (GA) [12], Teaching–Learning-Based Optimization (TLBO) [15], the Gravitational Search Algorithm (GSA) [14], the Whale Optimization Algorithm (WOA) [17], the Grey Wolf Optimizer (GWO) [16], the Tunicate Swarm Algorithm (TSA) [19], and the Marine Predators Algorithm (MPA) [18]. The simulation results of the performance and implementation of the TOA and compared optimization algorithms on the mentioned twenty-three objective functions are shown using two indicators of the average of the obtained best solutions (ave.) and the standard deviation of the obtained best solutions (std.).

The values of the parameters are selected based on the values used in similar studies and those proposed by the main authors of the algorithms. In general, having control parameters is a negative point for optimization algorithms. In fact, setting the appropriate values for the control parameters of optimization algorithms is a major challenge that has a significant impact on the performance of optimization algorithms. However, standard values for control parameters are usually suggested by algorithm designers. The values used for the main controlling parameters of the comparative algorithms are specified in Table 2.

### 3.1. Evaluation Unimodal Objective Function

The set of objective functions $F_{1}$ to $F_{7}$ has been selected from the unimodal objective functions. The proposed TOA and eight compared optimization algorithms are applied to optimize these objective functions. The optimization results of these objective functions are presented in Table 3. Based on the results of this table, the TOA is able to provide the global optimal solutions for $F_{1}$, $F_{2}$, $F_{3}$, $F_{4}$, and $F_{6}$. The proposed TOA is also the best optimizer for objective functions $F_{5}$ and $F_{7}$ and has been able to provide better quasi-optimal solutions than the compared algorithms. The optimization results show that the TOA has provided a suitable and effective performance in solving this type of objective function and is much more competitive than the eight compared optimization algorithms.

### 3.2. Evaluation High-Dimensional Multimodal Objective Functions

The objective functions from $F_{8}$ to $F_{13}$ are high-dimensional multimodal functions. The results of the implementation of the TOA and eight comparative optimization algorithms on this type of optimization problem are presented in Table 4. According to this table, it is clear that for the functions $F_{9}$ and $F_{11}$, the proposed TOA provides the global optimal solutions. Analysis of the simulation results indicates the optimal ability of the proposed TOA to solve high-dimensional multimodal optimization problems.

### 3.3. Evaluation Fixed-Dimensional Multimodal Objective Functions

The set of objective functions from $F_{14}$ to $F_{23}$ has been selected from the fixed-dimensional multimodal objective functions to analyze the optimization algorithms. Table 5 presents the optimization results of this type of objective function using the TOA and eight compared optimization algorithms. Based on this table, the TOA is able to provide the global optimal for $F_{18}$. Although in other objective functions the index “ave.” obtained from the TOA is similar to some of the compared optimization algorithms, the TOA has a more efficient ability to solve this type of objective function by providing better performance in index “std.”. The optimization results show that the proposed TOA has a more efficient performance in solving this type of objective function.

**Figure 2:**
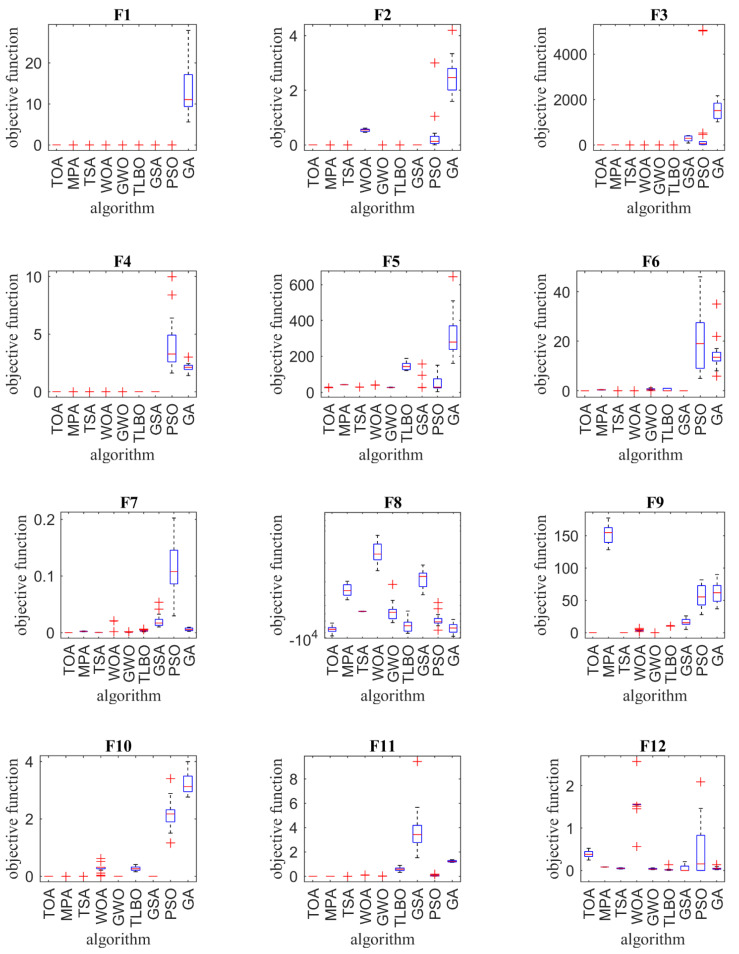

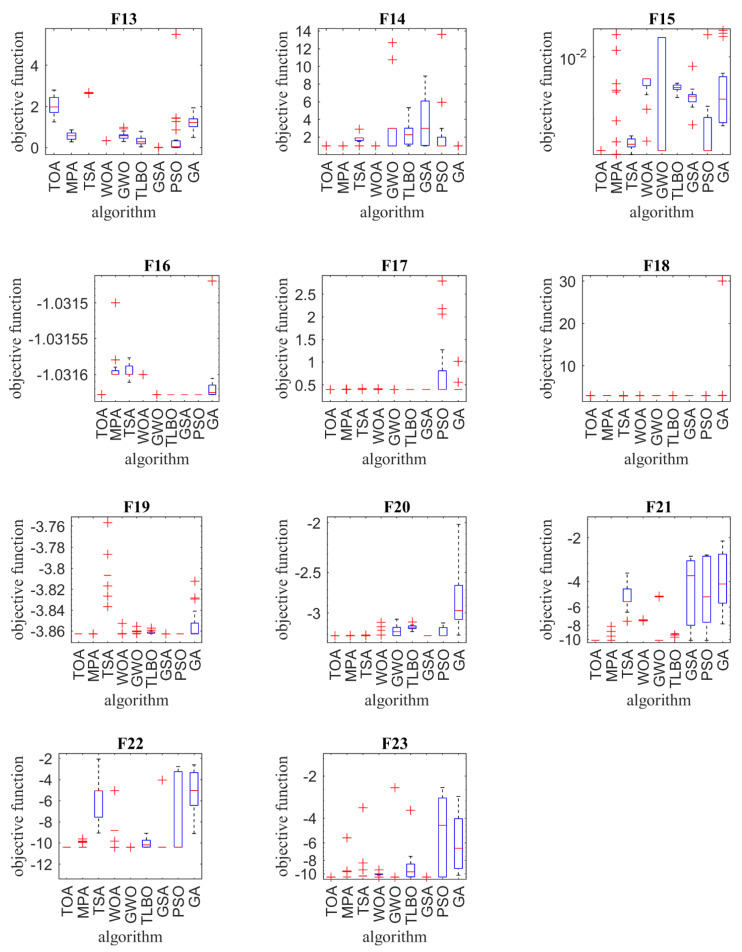
Boxplot of composition objective function results for different optimization algorithms.

In order to compare the performance of optimization algorithms in solving optimization problems, the optimization results of twenty-three objective functions using the proposed TOA as well as eight compared optimization algorithms are presented in the form of the boxplot in Figure 2.

### 3.4. Statistical Analysis

The results of the optimization of objective functions using two indexes of averages of the best obtained quasi-optimal solutions and their standard deviation provide important and valuable information about the ability and effectiveness of optimization algorithms. However, even with a very low probability, the superiority of one optimization algorithm over the compared optimization algorithms may be stochastic after twenty independent runs. Therefore, a statistical test is used in order to ensure the superiority of the proposed TOA over the eight compared optimization algorithms. In this regard, the non-parametric Wilcoxon rank sum test is applied. The Wilcoxon rank sum test is used to evaluate the similarity of two dependent samples with a ranking scale.

A *p*-value specifies whether the given algorithm is statistically significant or not. If the *p*-value of the given optimization algorithm is less than 0.05, then the corresponding optimization algorithm is statistically significant. The result of the analysis using the Wilcoxon rank test for the objective functions is shown in Table 6. It can be observed from Table 6 that the TOA is significantly superior to the eight compared optimization algorithms based on the *p*-values, which are less than 0.05.

In addition, in order to further analyze the results and performance of the optimization algorithms, another test, called the Friedman rank test, is used. The results of this test are presented in Table 7. Based on the results of the Friedman test, the proposed TOA ranks first in optimizing of all three types of unimodal, high-dimensional multimodal, and fixed-dimensional multimodal objective functions compared to the GA, PSO, GSA, TLBO, GWO, WOA, TSA, and MPA.

### 3.5. Sensitivity Analysis

In this section, the effects of two important parameters, the number of members of the algorithm population and the maximum number of iterations, on the performance of the proposed TOA in optimizing the objective functions are analyzed.

**Figure 3:**
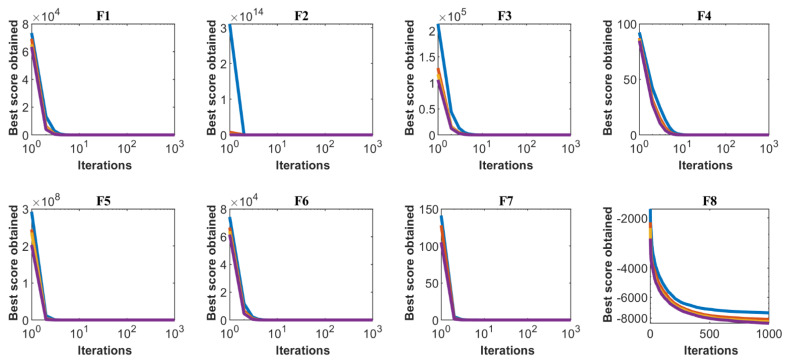

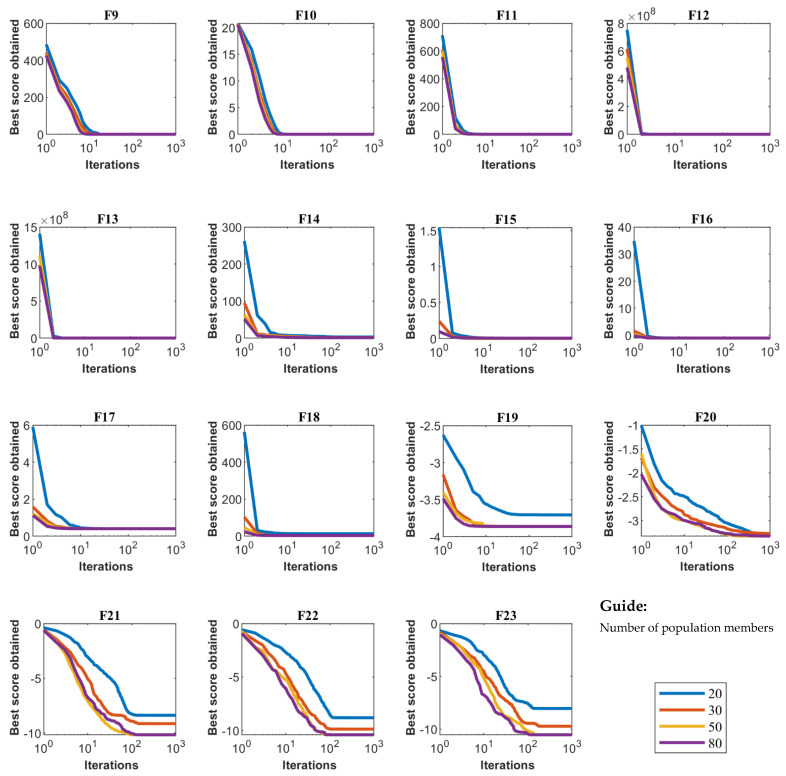
Sensitivity analysis of TOA for number of population members.

Sensitivity analysis of the proposed TOA to the parameter of the number of members of the population matrix is carried out for all twenty-three standard objective functions for numbers of members of the population of 20, 30, 50, and 80. The results of the sensitivity analysis of the TOA to the number of population members are presented in Table 8. Based on the analysis of the results presented in this table, it is found that when increasing the number of population members, the value of the objective function is decreased. Additionally, the effect of population members on the behavior of convergence curves is presented in Figure 3.

**Figure 4:**
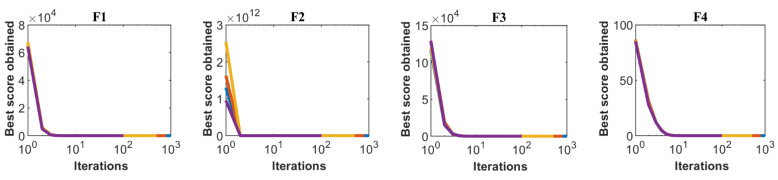

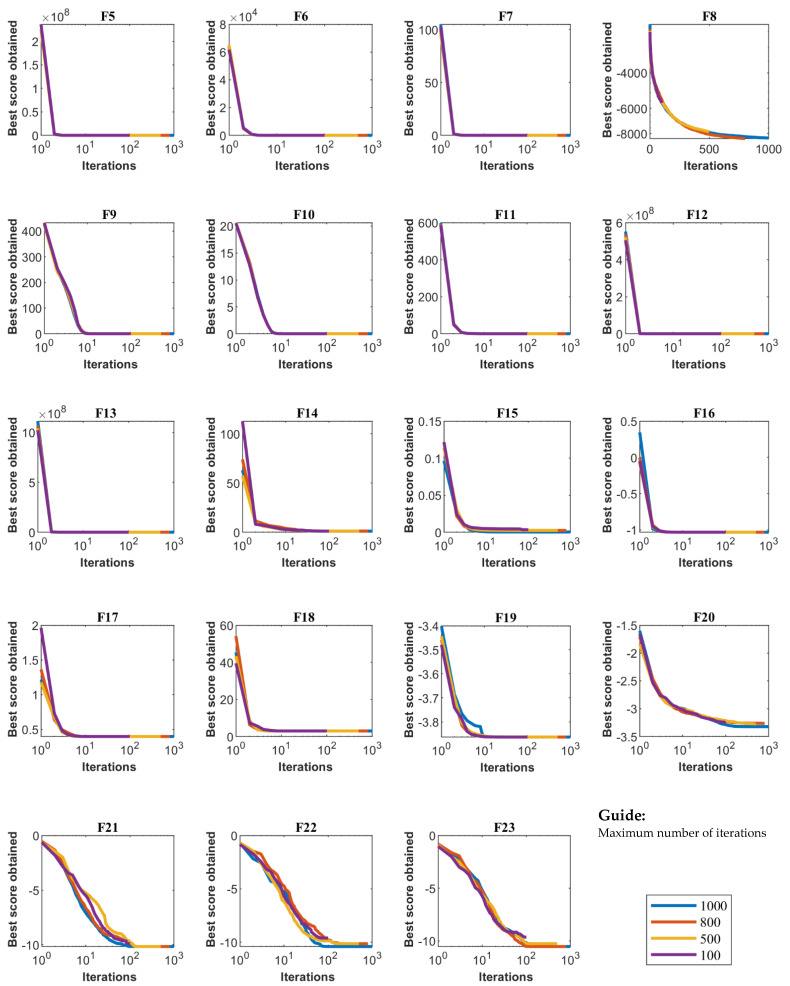
Sensitivity analysis of TOA for maximum number of iterations.

Sensitivity analysis of the TOA for the maximum number of iterations of the algorithm is carried out for all twenty-three standard objective functions for numbers of iterations of 100, 500, 800, and 100 repetitions. The simulation results obtained from this analysis for all objective functions and for the number of different iterations are presented in Table 9. Based on the sensitivity analysis of the number of iterations of the algorithm, it has been shown that by increasing the maximum number of iterations of the algorithm, the values of the objective functions are reduced and more suitable quasi-optimal solutions are obtained. The convergence curves of the objective functio1ns are plotted in Figure 4 under the influence of the different maximum numbers of iterations.

## 4. Discussion

Exploitation and exploration indicators are two important and effective criteria in evaluating and analyzing the performance of optimization algorithms and comparing them with each other.

The exploitation index is an important criterion that indicates the ability of the algorithm to achieve a suitable quasi-optimal solution. In fact, according to the concept of exploitation, an optimization algorithm must be able to provide a quasi-optimal solution after a complete implementation of an optimization problem. Therefore, in comparing the exploitation index between several different algorithms, an algorithm has a higher ability in the exploitation index which can provide a more suitable quasi-optimal solution. The objective functions of the unimodal type, including $F_{1}$ to $F_{7}$, have only one basic optimal solution. For this reason, these types of functions are desirable in order to evaluate the exploitation index. Based on the optimization results of these objective functions, as shown in Table 3, the proposed TOA has been able to achieve the global optimal solutions in the objective functions of $F_{1}$, $F_{2}$, $F_{3}$, $F_{4}$, and $F_{6}$. Additionally, the TOA in $F_{5}$ and $F_{7}$ functions has provided a more suitable quasi-optimal solution than similar algorithms. These results indicate the high ability of the TOA in the exploitation index and the presentation of quasi-optimal solutions.

The exploration index is another criterion in evaluating the performance of optimization algorithms, which shows the ability of the algorithm to accurately search the problem-solving space and not get caught up in local optimal solutions. In fact, according to the concept of exploration, an optimization algorithm should be able to search the various regions of the problem-solving space during successive iterations of the algorithm and provide a suitable quasi-optimal solution that is close to the global optimal one. High-dimensional multimodal objective functions, including $F_{8}$ to $F_{13}$, as well as fixed-dimensional multimodal objective functions, including $F_{14}$ to $F_{23}$, have several local optimal solutions, and achieving the global optimal solution of these functions is a challenge. Therefore, these objective functions are suitable for evaluating the exploration index of optimization algorithms. Based on the optimization results of these objective functions, presented in Table 4 and Table 5, the TOA in the objective functions $F_{9}$, $F_{11}$, and $F_{15}$ has been able to provide the global optimal with its high exploration ability. Additionally, the TOA has provided acceptable performance in other objective functions and has been able to provide appropriate quasi-optimal solutions by carefully searching the problem-solving space. The results of optimizing the set of multimodal objective functions show that the proposed TOA has a high ability in the index of exploration and accurate search of the problem-solving space.

The important thing about optimization algorithms as stochastic methods is that it cannot be claimed that a particular algorithm provides the best performance in all optimization problems. It is also possible to improve the performance of an algorithm by modifying it. In this study, the proposed TOA is compared with standard versions of the GA, PSO, GSA, TLBO, GWO, WOA, TSA, and MPA. However, the results obtained from the proposed TOA are far more competitive than the results of the implementation of the eight algorithms. The TOA has also been able to provide optimal global solutions in $F_{1}$, $F_{2}$, $F_{3}$, $F_{4}$, $F_{6}$, $F_{9}$, $F_{11}$, and $F_{18}$ functions. Therefore, one of the pros of the proposed TOA is that it is able to offer much more competitive solutions than similar algorithms. On the other hand, one of the cons of any optimization algorithm is that new optimization algorithms may be designed in the future that provide quasi-optimal solutions that are more appropriate and closer to the global optimal.

A comparative review of the TOA and compared algorithms, contrasting the characteristics of the heuristics, is presented in Table 10.

### Comparison with Blackboard System

Based on the theoretical comparison of the TOA and Blackboard system, one can understand that it is very difficult to compare the two algorithms in the same conditions. The main idea in the Blackboard system is based on the metaphor of a group of experts standing next to a large blackboard to solve a problem. The blackboard is actually a place to develop a solution to the problem. Every specialist is waiting for the suitable time to use the blackboard and improve the solution [11]. However, the main idea of the proposed TOA is the hierarchical structure of management in teamwork, consisting of a supervisor and other team members.

In the structure of the proposed TOA, the supervisor leads the team members to achieve a specific goal. In fact, the team members are influenced by the supervisor’s information and even other better members of the population to update the proposed solutions. However, in the Blackboard structure, instead of the population of members, there is a set of knowledge modules named the Knowledge System (KS) and the KS does not use any other KSs to improve participation in problem solving over time.

In the TOA, the number of population members is constant during execution and problem solving, while in the Blackboard system, without changing other KSs, additional KSs can be added to the Blackboard system, inappropriate KSs can be removed, and poorer performing KSs can be enhanced.

From the analysis of the above concepts, it can be deduced that although there are some similarities in the idea of the proposed TOA and the Blackboard system, they differ completely in structure, updating, and implementation.

## 5. Conclusions and Future Works

There are many optimization problems in different disciplines of science that should be optimized using appropriate methods. Population optimization algorithms are one of the most efficient and widely used methods to solve optimization problems. In this paper, a new optimization algorithm called the Teamwork Optimization Algorithm (TOA) has been described. The main idea in designing the proposed TOA was to model the relationships and teamwork behaviors of the members of a team in order to achieve the goal of that team. In teamwork, the supervisor is the member who performs best and is responsible for leading the team. Other people carry out their activities as team members under the supervision of a supervisor. Each team member tries to improve their performance by being influenced by the supervisor’s instructions as well as following the example of other team members who perform better than themselves. Additionally, each team member tries to improve his/her situation based on his/her individual activities in order to have a greater share in the team’s achievement of the goal. The theory of the TOA was described and then mathematically modeled for implementation in solving optimization problems. The quality of the TOA in solving optimization problems has been tested on twenty-three standard objective functions of unimodal, high-dimensional multimodal, and fixed-dimensional multimodal types. The results showed that the proposed TOA has a good ability to provide quasi-optimal solutions. Additionally, the performance of the TOA is compared with eight well-known optimization algorithms in providing quasi-optimal solutions. Analysis and evaluation of the results showed that the proposed TOA performed better in optimizing the objective functions and is much more competitive than the eight compared optimization algorithms.

The authors suggest some ideas and perspectives for future studies. The design of the binary version as well as the multiobjective version of the TOA is an interesting possibility for future investigations. Apart from this, implementing the TOA on various optimization problems and real-world optimization problems can be considered as significant contributions, as well. The use of a collaborative learning phase as a corrective phase in improving the performance of other algorithms is also a suggestion for further studies.

## Figures and Tables

**Table 1:** The various steps of the proposed TOA for the first iteration in sphere function solving.

	Step 1		Step 2	Step 3	Step 4		Step 5				Step 6			Final
	X		*F(X)*	*S*	*X*		*X^M^*		*X*		*X*		*F(X)*	
	$x_{1}$	$x_{2}$			$x_{1}$	$x_{2}$	$x_{1}^{M}$	$x_{2}^{M}$	$x_{1}$	$x_{2}$	$x_{1}$	$x_{2}$		
$X_{1}$	98.12666	72.68003	14911.23		19.65399	20.55081	14.63504	17.21673	17.16322	8.697356	17.16322	8.697356	370.22	3.97 × 10^−55^
$X_{2}$	37.61062	17.84777	1733.102		29.87663	11.62747	15.89913	12.95704	21.92269	5.767422	21.92269	5.767422	513.8675	8.85 × 10^−56^
$X_{3}$	19.00723	−69.7136	5221.263		10.5817	−38.3855	17.90698	10.5605	10.17539	−32.2784	10.17539	−32.2784	1145.432	1.84 × 10^−57^
$X_{4}$	51.43423	49.23254	5069.323		25.26888	26.46832	15.97408	−0.14922	24.50555	22.1087	24.50555	22.1087	1089.317	1.71 × 10^−57^
$X_{5}$	14.63504	17.21673	510.6002	$X_{5}$	12.52158	17.21673	17.16322	8.697356	9.967937	14.45587	9.967937	14.45587	308.332	4.15 × 10^−57^
$X_{6}$	59.62064	51.23127	6179.263		52.33316	48.86162	22.99721	0.240222	−22.3172	4.418826	−22.3172	4.418826	517.582	1.35 × 10^−55^
$X_{7}$	84.65901	−87.3821	14802.78		32.22874	56.36313	16.52373	0.837165	7.995059	−0.20421	7.995059	−0.20421	63.96267	6.64 × 10^−54^
$X_{8}$	54.2485	−17.3096	3242.523		45.23606	−5.51647	9.916097	3.280799	19.61827	0.864246	19.61827	0.864246	385.6233	1.88 × 10^−56^
$X_{9}$	−46.1119	77.9884	8208.503		−35.6225	67.49466	11.12887	2.97873	−5.91138	19.88028	−5.91138	19.88028	430.1698	5.42 × 10^−58^
$X_{10}$	89.51302	75.22706	13671.69		77.52489	65.93948	9.235507	4.85668	56.40553	47.04885	56.40553	47.04885	5395.179	9.51 × 10^−56^
Best Solution: $x_{1}=-$2.0213 × 10^−29^, $x_{2}=-$1.1566 × 10^−29^ and F(X)= 5.4232 × 10^−58^														

**Table 2:** Parameter values for the comparative algorithms.

Algorithm	Parameter	Value
GA		
	Type	Real coded
	Selection	Roulette wheel (proportionate)
	Crossover	Whole arithmetic (probability = 0.8, α∈[−0.5, 1.5])
	Mutation	Gaussian (probability = 0.05)
PSO		
	Topology	Fully connected
	Cognitive and social constant	$C_{1}=2,C_{2}=2$
	Inertia weight	Linear reduction from 0.9 to 0.1
	Velocity limit	10% of dimension range
GSA		
	Alpha, $G_{0}$, $R_{norm}$, $R_{power}$	20, 100, 2, 1
TLBO		
	$T_{F}$: teaching factor	$T_{F}=$ round [(1+rand)]
	Random number	*rand* is a random number in [0, 1].
GWO		
	Convergence parameter (*a*)	*a*: Linear reduction from 2 to 0.
WOA		
	Convergence parameter (*a*)	*a*: Linear reduction from 2 to 0.
	*r* is a random vector in [0, 1].	
	*l* is a random number in [−1, 1].	
TSA		
	$P_{min}$ and $P_{max}$	1, 4
	$c_{1},\;c_{2},c_{3}$	Random numbers lie in the range of [0–1].
MPA		
	Constant number	*P* = 0.5
	Random vector	*R* is a vector of uniform random numbers in [0, 1].
	Fish Aggregating Devices (*FADs*)	*FADs* = 0.2
	Binary vector	*U* = 0 or 1

**Table 3:** Optimization results of TOA and compared algorithms on unimodal test function.

		MPA	TSA	WOA	GWO	TLBO	GSA	PSO	GA	TOA
$F_{1}$	Ave	3.2715 × 10^−21^	7.71 × 10^−38^	2.1741 × 10^−9^	1.09 × 10^−58^	8.3373 × 10^−60^	2.0255 × 10^−17^	1.7740 × 10^−5^	13.2405	0
	std	4.6153 × 10^−21^	7.00 × 10^−21^	7.3985 × 10^−25^	5.1413 × 10^−74^	4.9436 × 10^−76^	1.1369 × 10^−32^	6.4396 × 10^−21^	4.7664 × 10^−15^	0
$F_{2}$	Ave	1.57 × 10^−12^	8.48 × 10^−39^	0.5462	1.2952 × 10^−34^	7.1704 × 10^−35^	2.3702 × 10^−8^	0.3411	2.4794	0
	std	1.42 × 10^−12^	5.92 × 10^−41^	1.7377 × 10^−16^	1.9127 × 10^−50^	6.6936 × 10^−50^	5.1789 × 10^−24^	7.4476 × 10^−17^	2.2342 × 10^−15^	0
$F_{3}$	Ave	0.0864	1.15 × 10^−21^	1.7634 × 10^−8^	7.4091 × 10^−15^	2.7531 × 10^−15^	279.3439	589.492	1536.8963	0
	std	0.1444	6.70 × 10^−21^	1.0357 × 10^−23^	5.6446 × 10^−30^	2.6459 × 10^−31^	1.2075 × 10^−13^	7.1179 × 10^−13^	6.6095 × 10^−13^	0
$F_{4}$	Ave	2.6 × 10^−8^	1.33 × 10^−23^	2.9009 × 10^−5^	1.2599 × 10^−14^	9.4199 × 10^−15^	3.2547 × 10^−9^	3.9634	2.0942	0
	std	9.25 × 10^−9^	1.15 × 10^−22^	1.2121 × 10^−20^	1.0583 × 10^−29^	2.1167 × 10^−30^	2.0346 × 10^−24^	1.9860 × 10^−16^	2.2342 × 10^−15^	0
$F_{5}$	Ave	46.049	28.8615	41.7767	26.8607	146.4564	36.10695	50.26245	310.4273	26.2476
	std	0.4219	4.76 × 10^−3^	2.5421 × 10^−14^	0	1.9065 × 10^−14^	3.0982 × 10^−14^	1.5888 × 10^−14^	2.0972 × 10^−13^	3.26× 10^−14^
$F_{6}$	Ave	0.398	7.10 × 10^−21^	1.6085 × 10^−9^	0.6423	0.4435	0	20.25	14.55	0
	std	0.1914	1.12 × 10^−25^	4.6240 × 10^−25^	6.2063 × 10^−17^	4.2203 × 10^−16^	0	1.2564	3.1776 × 10^−15^	0
$F_{7}$	Ave	0.0018	3.72 × 10^−4^	0.0205	0.0008	0.0017	0.0206	0.1134	5.6799 × 10^−3^	9.92× 10^−6^
	std	0.001	5.09 × 10^−5^	1.5515 × 10^−18^	7.2730 × 10^−20^	3.8789 × 10^−19^	2.7152 × 10^−18^	4.3444 × 10^−17^	7.7579 × 10^−19^	1.74 × 10^−20^

**Table 4:** Optimization results of TOA and compared algorithms on high-dimensional test function.

		MPA	TSA	WOA	GWO	TLBO	GSA	PSO	GA	TOA
$F_{8}$	Ave	−3594.16321	−5740.3388	−1663.9782	−5885.1172	−7408.6107	−2849.0724	−6908.6558	−8184.4142	−9631.41
	std	811.32651	41.5	716.3492	467.5138	513.5784	264.3516	625.6248	833.2165	3.86 × 10^−12^
$F_{9}$	Ave	140.1238	5.70 × 10^−3^	4.2011	8.5265 × 10^−15^	10.2485	16.2675	57.0613	62.4114	0
	std	26.3124	1.46 × 10^−3^	4.3692 × 10^−15^	5.6446 × 10^−30^	5.5608 × 10^−15^	3.1776 × 10^−15^	6.3552 × 10^−15^	2.5421 × 10^−14^	0
$F_{10}$	Ave	9.6987 × 10^−12^	9.80 × 10^−14^	0.3293	1.7053 × 10^−14^	0.2757	3.5673 × 10^−9^	2.1546	3.2218	8.88 × 10^−16^
	std	6.1325 × 10^−12^	4.51 × 10^−12^	1.9860 × 10^−16^	2.7517 × 10^−29^	2.5641 × 10^−15^	3.6992 × 10^−25^	7.9441 × 10^−16^	5.1636 × 10^−15^	0
$F_{11}$	Ave	0	1.00 × 10^−7^	0.1189	0.0037	0.6082	3.7375	0.0462	1.2302	0
	std	0	7.46 × 10^−7^	8.9991 × 10^−17^	1.2606 × 10^−18^	1.9860 × 10^−16^	2.7804 × 10^−15^	3.1031 × 10^−18^	8.4406 × 10^−16^	0
$F_{12}$	Ave	0.0851	0.0368	1.7414	0.0372	0.0203	0.0362	0.4806	0.047	0.2463
	std	0.0052	1.5461 × 10^−2^	8.1347 × 10^−12^	4.3444 × 10^−17^	7.7579 × 10^−19^	6.2063 × 10^−18^	1.8619 × 10^−16^	4.6547 × 10^−18^	7.45 × 10^−17^
$F_{13}$	Ave	0.4901	2.9575	0.3456	0.5763	0.3293	0.002	0.5084	1.2085	1.25
	std	0.1932	1.5682 × 10^−12^	3.2539 × 10^−12^	2.4825 × 10^−16^	2.1101 × 10^−16^	4.2617 × 10^−14^	4.9650 × 10^−17^	3.2272 × 10^−16^	4.47 × 10^−16^

**Table 5:** Optimization results of TOA and compared algorithms on fixed-dimensional test function.

		MPA	TSA	WOA	GWO	TLBO	GSA	PSO	GA	TOA
$F_{14}$	Ave	0.998	1.9923	0.998	3.7408	2.2721	3.5913	2.1735	0.9986	0.998
	std	4.2735 × 10^−16^	2.6548 × 10^−7^	9.4336 × 10^−16^	6.4545 × 10^−15^	1.9860 × 10^−16^	7.9441 × 10^−16^	7.9441 × 10^−16^	1.5640 × 10^−15^	4.72 × 10^−16^
$F_{15}$	Ave	0.003	0.0004	0.0049	0.0063	0.0033	0.0024	0.0535	5.3952 × 10^−2^	0.0003
	std	4.0951 × 10^−15^	9.0125 × 10^−4^	3.4910 × 10^−18^	1.1636 × 10^−18^	1.2218 × 10^−17^	2.9092 × 10^−19^	3.8789 × 10^−19^	7.0791 × 10^−18^	1.16 × 10^−18^
$F_{16}$	Ave	−1.0316	−1.0316	−1.0316	−1.0316	−1.0316	−1.0316	−1.0316	−1.0316	−1.0316
	std	4.4652 × 10^−16^	2.6514 × 10^−16^	9.9301 × 10^−16^	3.9720 × 10^−16^	1.4398 × 10^−15^	5.9580 × 10^−16^	3.4755 × 10^−16^	7.9441 × 10^−16^	1.99 × 10^−16^
$F_{17}$	Ave	0.3979	0.3991	0.4047	0.3978	0.3978	0.3978	0.7854	0.4369	0.3978
	std	9.1235 × 10^−15^	2.1596 × 10^−16^	2.4825 × 10^−17^	8.6888 × 10^−17^	7.4476 × 10^−17^	9.9301 × 10^−17^	4.9650 × 10^−17^	4.9650 × 10^−17^	9.93 × 10^−17^
$F_{18}$	Ave	3	3	3	3	3.0009	3	3	4.3592	3
	std	1.9584 × 10^−15^	2.6528 × 10^−15^	5.6984 × 10^−15^	2.0853 × 10^−15^	1.5888 × 10^−15^	6.9511 × 10^−16^	3.6741 × 10^−15^	5.9580 × 10^−16^	0
$F_{19}$	Ave	−3.8627	−3.8066	−3.8627	−3.8621	−3.8609	−3.8627	−3.8627	−3.85434	−3.8628
	std	4.2428 × 10^−15^	2.6357 × 10^−15^	3.1916 × 10^−15^	2.4825 × 10^−15^	7.3483 × 10^−15^	8.3413 × 10^−15^	8.9371 × 10^−15^	9.9301 × 10^−17^	2.68 × 10^−16^
$F_{20}$	Ave	−3.3211	−3.3206	−3.2424	−3.2523	−3.2014	−3.0396	−3.2619	−2.8239	−3.322
	std	1.1421 × 10^−11^	5.6918 × 10^−15^	7.9441 × 10^−16^	2.1846 × 10^−15^	1.7874 × 10^−15^	2.1846 × 10^−14^	2.9790 × 10^−16^	3.9720 × 10^−16^	1.69 × 10^−15^
$F_{21}$	Ave	−10.1532	−5.5021	−7.4016	−9.6452	−9.1746	−5.1486	−5.3891	−4.3040	−10.1532
	std	2.5361 × 10^−11^	5.4615 × 10^−13^	2.3819 × 10^−11^	6.5538 × 10^−15^	8.5399 × 10^−15^	2.9790 × 10^−16^	1.4895 × 10^−15^	1.5888 × 10^−15^	1.39 × 10^−15^
$F_{22}$	Ave	−10.4029	−5.0625	−8.8165	−10.4025	−10.0389	−9.0239	−7.6323	−5.1174	−10.4029
	std	2.8154 × 10^−11^	8.4637 × 10^−14^	6.7524 × 10^−15^	1.9860 × 10^−15^	1.5292 × 10^−14^	1.6484 × 10^−12^	1.5888 × 10^−15^	1.2909 × 10^−15^	3.18 × 10^−15^
$F_{23}$	Ave	−10.5364	−10.3613	−10.0003	−10.1302	−9.2905	−8.9045	−6.1648	−6.5621	−10.5364
	std	3.9861 × 10^−11^	7.6492 × 10^−12^	9.1357 × 10^−15^	4.5678 × 10^−15^	1.1916 × 10^−15^	7.1497 × 10^−14^	2.7804 × 10^−15^	3.8727 × 10^−15^	7.94 × 10^−16^

**Table 6:** Obtained results from the Wilcoxon test (*p* ≥ 0.05).

Compared Algorithms	Unimodal	High-Multimodal	Fixed-Multimodal
TOA vs. MPA	0.015625	0.625	0.125
TOA vs. TSA	0.015625	0.21875	0.007813
TOA vs. WOA	0.015625	0.15625	0.015625
TOA vs. GWO	0.015625	0.84375	0.015625
TOA vs. TLBO	0.015625	0.3125	0.007813
TOA vs. GSA	0.03125	0.3125	0.015625
TOA vs. PSO	0.015625	0.15625	0.007813
TOA vs. GA	0.015625	0.15625	0.003906

**Table 7:** Results of the Friedman rank test.

Test Function		GA	PSO	GSA	TLBO	GWO	WOA	TSA	MPA	TOA
Unimodal ($F_{1}-F_{7}$)	Friedman value	57	56	37	28	26	42	37	17	7
	Friedman rank	8	7	5	4	3	6	5	2	1
High-dimensional multimodal ($F_{8}-F_{13}$)	Friedman value	38	36	30	23	22	37	31	26	19
	Friedman rank	9	7	5	3	2	8	6	4	1
Fixed-dimensional multimodal ($F_{14}-F_{23}$)	Friedman value	56	46	39	36	32	34	16	34	10
	Friedman rank	8	7	6	5	3	4	2	4	1
All 23 test functions	Friedman value	151	138	106	87	80	113	84	77	36
	Friedman rank	9	8	6	5	3	7	4	2	1

**Table 8:** Results of the algorithm sensitivity analysis of the number of population members.

Objective Function	Number of Population Members			
	20	30	50	80
$F_{1}$	0	0	0	0
$F_{2}$	0	0	0	0
$F_{3}$	0	0	0	0
$F_{4}$	0	0	0	0
$F_{5}$	28.0905	27.66011	26.24746	26.22656
$F_{6}$	0	0	0	0
$F_{7}$	4.49 × 10^−5^	2.18 × 10^−5^	9.92 × 10^−6^	2.87 × 10^−6^
$F_{8}$	−9222.78	−9279.11	−9631.41	−10035.1
$F_{9}$	0	0	0	0
$F_{10}$	8.88× 10^−16^	8.88× 10^−16^	8.88× 10^−16^	8.88× 10^−16^
$F_{11}$	0	0	0	0
$F_{12}$	0.367779	0.25017	0.246392	0.213644
$F_{13}$	1.7093	1.436574	1.25003	1.129791
$F_{14}$	0.998004	0.998004	0.998004	0.998004
$F_{15}$	0.000307	0.000307	0.000307	0.000307
$F_{16}$	−1.03163	−1.03163	−1.03163	−1.03163
$F_{17}$	0.397887	0.397887	0.397887	0.397887
$F_{18}$	3	3	3	3
$F_{19}$	−3.86278	−3.86278	−3.86278	−3.86278
$F_{20}$	−3.32199	−3.32199	−3.322	−3.322
$F_{21}$	−10.1529	−10.1532	−10.1532	−10.1532
$F_{22}$	−10.4028	−10.4029	−10.4029	−10.4029
$F_{23}$	−10.5363	−10.5364	−10.5364	−10.5364

**Table 9:** Results of the algorithm sensitivity analysis of the maximum number of iterations.

Objective Function	Maximum Number of Iterations			
	100	500	800	1000
*F*_1_	9.45 × 10^−90^	0	0	0
*F*_2_	9.19 × 10^−47^	1.5 × 10^−238^	0	0
$F_{3}$	1.83 × 10^−53^	1 × 10^−267^	0	0
$F_{4}$	1.68 × 10^−42^	2.5 × 10^−217^	0	0
$F_{5}$	28.0631	27.2074	27.20696	26.24746
$F_{6}$	0	0	0	0
$F_{7}$	0.000146	1.3 × 10^−5^	1.23 × 10^−5^	9.92 × 10^−6^
$F_{8}$	−6711.59	−8944.49	−9358.76	−9631.41
$F_{9}$	0	0	0	0
$F_{10}$	4.44 × 10^−15^	8.88 × 10^−16^	8.88 × 10^−16^	8.88 × 10^−16^
$F_{11}$	0	0	0	0
$F_{12}$	0.307775	0.301889	0.266274	0.246392
$F_{13}$	1.867947	1.59195	1.310176	1.25003
$F_{14}$	0.998004	0.998004	0.998004	0.998004
$F_{15}$	0.000307	0.000307	0.000307	0.000307
$F_{16}$	−1.03163	−1.03163	−1.03163	−1.03163
$F_{17}$	0.397887	0.397887	0.397887	0.397887
$F_{18}$	3	3	3	3
$F_{19}$	−3.86278	−3.86278	−3.86278	−3.86278
$F_{20}$	−3.32155	−3.32198	−3.32199	−3.322
$F_{21}$	−10.1532	−10.1532	−10.1532	−10.1532
$F_{22}$	−10.4029	−10.4029	−10.4029	−10.4029
$F_{23}$	−10.5364	−10.5364	−10.5364	−10.5364

**Table 10:** Comparative review of TOA and compared algorithms.

Algorithm	Advantage	Disadvantage
GA	Good global search, simplicity, and comprehensibility	High memory consumption, control parameters, and poor local search.
PSO	Simplicity of the relationship and its implementation.	Control parameters, poor convergence, and entrapment in local optimum areas.
GSA	Easy implementation, fast convergence in simple problems, and low computational cost.	High computation, time consuming, several control parameters, and poor convergence in complex objective functions.
TLBO	Good global search, simplicity, and no requirement for any parameters	Poor convergence rate.
GWO	Fast convergence due to continuous reduction of search space, fewer storage and computational requirements, and easily implemented due to its simple structure.	Low convergence speed, poor local search, and low accuracy in solving complex problems.
WOA	Simple structure, fewer required operators, and appropriate balance between exploration and exploitation.	Low accuracy, slow convergence, and easily falls into local optimum.
MPA	Good global search and fast convergence.	High computation, time consuming, and control parameters.
TSA	Fast convergence, good global search, and appropriate balance between exploration and exploitation.	Poor convergence, control parameters, and falling into local optimal solutions when solving high-dimensional multimodal problems.
TOA	Simplicity of equations, easy implementation, lack of control parameters, proper exploitation, proper exploration, not caught up in local optimal solutions, and high convergence power.	The important thing about all optimization algorithms is that it cannot be claimed that one particular algorithm is the best optimizer for all optimization problems. It is also always possible to develop new optimization algorithms that can provide more desirable quasi-optimal solutions that are also closer to the global optimal.

## Data Availability

The data present in this study are available on request from the author M.D.

## Appendix A

Information of the twenty-three objective functions is provided in Table A1, Table A2 and Table A3; these functions were introduced and studied by Yao [20]. Unimodal functions have only one optimal point and, in fact, the behavior of this type of function is such that if up to a point (mode) it is uniform and descending, from that point onwards, it is uniform but ascending. Multimodal functions, in addition to the main solution, also have several local solutions, and the main challenge in optimizing this type of function is not to get stuck in local solutions and approach the main solution.

**Table A1:** Unimodal test functions.

Objective Function	Range	Dimensions	F_min_
$F_{1}\left(x\right)=\overset{m}{\underset{i=1}{\sum }}x_{i}^{2}$	[−100, 100]	30	0
$F_{2}\left(x\right)=\overset{m}{\underset{i=1}{\sum }}\left|x_{i}\right|+\overset{m}{\underset{i=1}{\prod }}\left|x_{i}\right|$	[−10, 10]	30	0
$F_{3}\left(x\right)=\overset{m}{\underset{i=1}{\sum }}{\left(\overset{i}{\underset{j=1}{\sum }}x_{i}\right)}^{2}$	[−100, 100]	30	0
$F_{4}\left(x\right)=max\left\{\left|x_{i}\right|\;,\;\;1\leq i\leq m\;\right\}$	[−100, 100]	30	0
$F_{5}\left(x\right)=\overset{m-1}{\underset{i=1}{\sum }}\left[100{\left(x_{i+1}-x_{i}^{2}\right)}^{2}+{\left(x_{i}-1\right)}^{2})\right]$	[−30, 30]	30	0
$F_{6}\left(x\right)=\overset{m}{\underset{i=1}{\sum }}{\left(\left[x_{i}+0.5\right]\right)}^{2}$	[−100, 100]	30	0
$F_{7}\left(x\right)=\overset{m}{\underset{i=1}{\sum }}ix_{i}^{4}+random(0,1)$	[−1.28, 1.28]	30	0

**Table A2:** High-dimensional multimodal test functions.

Objective Function	Range	Dimensions	F_min_
$F_{8}\left(x\right)=\overset{m}{\underset{i=1}{\sum }}-x_{i}\;\sin\left(\sqrt{\left|x_{i}\right|}\right)$	[−500, 500]	30	−12,569
$F_{9}\left(x\right)=\overset{m}{\underset{i=1}{\sum }}\left[\;x_{i}^{2}-10\cos\left(2\pi x_{i}\right)+10\right]$	[−5.12, 5.12]	30	0
$F_{10}\left(x\right)=-20\exp(-0.2\sqrt{\frac{1}{m}\overset{m}{\underset{i=1}{\sum }}x_{i}^{2}})-\exp(\frac{1}{m}\overset{m}{\underset{i=1}{\sum }}\cos(2\pi x_{i}))+20+e$	[−32, 32]	30	0
$F_{11}\left(x\right)=\frac{1}{4000}\overset{m}{\underset{i=1}{\sum }}x_{i}^{2}-\overset{m}{\underset{i=1}{\prod }}\cos\left(\frac{x_{i}}{\sqrt{i}}\right)+1$	[−600, 600]	30	0
$F_{12}\left(x\right)=\frac{\pi }{m}\;\left\{10\sin\left(\pi y_{1}\right)+\overset{m}{\underset{i=1}{\sum }}{\left(y_{i}-1\right)}^{2}\left[1+10\;\sin^{2}\left(\pi y_{i+1}\right)\right]+{\left(y_{n}-1\right)}^{2}\right\}+\overset{m}{\underset{i=1}{\sum }}u\left(x_{i},10,100,4\right)$ $u\left(x_{i},a,i,n\right)=\{\begin{array}{cc} k{\left(x_{i}-a\right)}^{n}, & x_{i}>-a;\\ 0, & -a<x_{i}<a\\ k{\left(-x_{i}-a\right)}^{n},\; & x_{i}<-a \end{array};$	[−50, 50]	30	0
$\begin{array}{l} F_{13}\left(x\right)=0.1\{\sin^{2}\left(3\pi x_{1}\right)\\ \;\;\;\;\;+\overset{m}{\underset{i=1}{\sum }}{\left(x_{i}-1\right)}^{2}\left[1+\sin^{2}\left(3\pi x_{i}+1\right)\right]+{\left(x_{n}-1\right)}^{2}\\ \;\;\;\;\;[1+\sin^{2}\left(2\pi x_{m}\right)]\}+\overset{m}{\underset{i=1}{\sum }}u\left(x_{i},5,100,4\right) \end{array}$	[−50, 50]	30	0

**Table A3:** Fixed-dimensional multimodal test functions.

Objective Function	Range	Dimensions	F_min_
$F_{14}\left(x\right)={\left(\frac{1}{500}+\overset{25}{\underset{j=1}{\sum }}\frac{1}{j+\sum_{i=1}^{2}{\left(x_{i}-a_{ij}\right)}^{6}}\right)}^{-1}$	[−65.53, 65.53]	2	0.998
$F_{15}\left(x\right)=\overset{11}{\underset{i=1}{\sum }}{\left[a_{i}-\frac{x_{1}\left(b_{i}^{2}+b_{i}x_{2}\right)}{b_{i}^{2}+b_{i}x_{3}+x_{4}}\right]}^{2}$	[−5, 5]	4	0.00030
$F_{16}\left(x\right)=4x_{1}^{2}-2.1x_{1}^{4}+\frac{1}{3}x_{1}^{6}+x_{1}x_{2}-4x_{2}^{2}+4x_{2}^{4}$	[−5, 5]	2	−1.0316
$F_{17}\left(x\right)={\left(x_{2}-\frac{5.1}{4\pi^{2}}x_{1}^{2}+\frac{5}{\pi }x_{1}-6\right)}^{2}+10\left(1-\frac{1}{8\pi }\right)\cos x_{1}+10$	[−5, 10] × [0, 15]	2	0.398
$\begin{array}{l} F_{18}\left(x\right)=\left[1+{\left(x_{1}+x_{2}+1\right)}^{2}\left(19-14x_{1}+3x_{1}^{2}-14x_{2}+6x_{1}x_{2}+3x_{2}^{2}\right)\right]\\ \;\;\;\;\times [30+{\left(2x_{1}-3x_{2}\right)}^{2}\times (18-32x_{1}+12x_{1}^{2}+48x_{2}\\ \;\;\;\;-36x_{1}x_{2}+27x_{2}^{2})] \end{array}$	[−5, 5]	2	3
$F_{19}\left(x\right)=-\overset{4}{\underset{i=1}{\sum }}c_{i}\exp\left(-\overset{3}{\underset{j=1}{\sum }}a_{ij}{\left(x_{j}-P_{ij}\right)}^{2}\right)$	[0, 1]	3	−3.86
$F_{20}\left(x\right)=-\overset{4}{\underset{i=1}{\sum }}c_{i}\exp\left(-\overset{6}{\underset{j=1}{\sum }}a_{ij}{\left(x_{j}-P_{ij}\right)}^{2}\right)$	[0, 1]	6	−3.22
$F_{21}\left(x\right)=-\overset{5}{\underset{i=1}{\sum }}{\left[\left(X-a_{i}\right){\left(X-a_{i}\right)}^{T}+6c_{i}\right]}^{-1}$	[0, 10]	4	−10.1532
$F_{22}\left(x\right)=-\overset{7}{\underset{i=1}{\sum }}{\left[\left(X-a_{i}\right){\left(X-a_{i}\right)}^{T}+6c_{i}\right]}^{-1}$	[0, 10]	4	−10.4029
$F_{23}\left(x\right)=-\overset{10}{\underset{i=1}{\sum }}{\left[\left(X-a_{i}\right){\left(X-a_{i}\right)}^{T}+6c_{i}\right]}^{-1}$	[0, 10]	4	−10.5364
